# Can Organizational Identification Weaken the Negative Effects of Customer Bullying?—Testing the Moderating Effect of Organizational Identification

**DOI:** 10.3389/fpsyg.2022.769087

**Published:** 2022-05-31

**Authors:** Haili Huang, Shengxian Yu, Pin Peng

**Affiliations:** ^1^School of Business Administration, Zhongnan University of Economics and Law, Wuhan, China; ^2^School of Business Administration, South China University of Technology, Guangzhou, China; ^3^School of Finance, Hunan University of Finance and Economics, Changsha, China

**Keywords:** customer bullying, job insecurity, organizational identification, unethical behaviors, conservation of resources theory

## Abstract

Customer bullying is a common phenomenon, causing short-term emotional distress or having long-term psychological impact on frontline employees of service enterprises, yielding either direct or indirect losses to service enterprises. While existing research has focused on the emotional and psychological impact of customer bullying on employees, little attention has been directed at the impact of customer bullying on negative employee behavior and internal mechanisms. In view of this, this paper draws on conservation of resources theory and discusses how and when customer bullying can lead to unethical behaviors. Furthermore, the mediating role of job insecurity and the moderating effect of organizational identification are analyzed. In study 1, 181 valid questionnaire data were collected at two time points, and regression data analysis was used to explore the effect of customer bullying on unethical behaviors through job insecurity. In study 2, 212 employees were recruited to investigate the moderating effect of organizational identification between customer bullying and unethical behaviors through a scenario experimental study. The results reveal that customer bullying is positively related to employees’ job insecurity and unethical behaviors. Job insecurity partially mediates the positive relationship between customer bullying and unethical behaviors. Further, the regression analysis results indicate that the direct effect of customer bullying on unethical behaviors is moderated by organizational identification. This study provides theoretical guidance for entrepreneurs to reduce both employee job insecurity and unethical behavior.

## Introduction

During service interactions, some customers frequently treat employees in an unkind, verbally abusive, violent, and loud manner (Harris and Reynolds, 2004). From the perspective of interpersonal interaction justice, customer bullying refers to “low-quality” interpersonal treatment that employees receive from customers during a service interaction with them (Zhan et al., 2015; Yue et al., 2017). A growing body of research showed that there is a dark side to the relationship between customer behaviors and front-line service employee responses (Rafaeli et al., 2012). Current research on this organizational dark side behavior focuses on workplace bullying within organizations, such as counterproductive behaviors and workplace bullying among colleagues or supervisors. Few studies have focused on behaviors from outside the organization, such as customer bullying. Existing research on customer bullying mostly focuses on theoretical discussions, and to a lesser extent on how to eliminate the negative impact of customer bullying on employees (Wen and Liang, 2020; Baek et al., 2022). In the intra-organizational context, Sarwar et al. (2020) confirmed that workplace bullying triggers job insecurity among nurses, thus leading to deviant behavior. However, the difference is that in the service situation, because interactions with customers occupy most employees’ work time, the bullying experienced by employees mainly originates from customers rather than colleagues or supervisors (Grandey et al., 2007). In other words, it is more common for frontline service workers to experience being bullied by customers from outside the organization than from colleagues or supervisors within the organization. It is worth noting that customer bullying mostly occurs in service industries that have close contact and frequent interactions with customers, e.g., hotels, catering, tourism, and other industries (Cheng et al., 2019). Because of the extent of COVID-19, restaurant and hotel industries are operating under great pressure, and companies are placing higher demands on their frontline service staff to improve the customer experience. It has been found to be more common for employees to suffer from customer bullying in a situation of power asymmetry between these two parties (Zhao and Liu, 2019). Moreover, Harvey et al. (2006) indicated that cultural factors significantly impact the perpetration of aggression. Compared to Western countries, the long-standing feudal authoritarianism and Confucian culture have led to the Chinese people’s notion of power and compliance with authority, which makes bullying behavior more prevalent in the workplace. Therefore, in the context of the Chinese culture, it is of theoretical and practical significance to explore the influence mechanism of customer bullying on employees’ work response.

This study introduces unethical behaviors as the outcome variable. In the service industry, negative employee behaviors triggered by customer bullying have become more frequent (Reynolds and Harris, 2006; He et al., 2019). Currently, only few studies have explored the impact of customer bullying on employee negative behavior from the perspective of employee destructive behavior (Jaarsveld et al., 2010; Jiang and Lin, 2021). However, in realistic interactive service contexts, many employee negative behaviors caused by customer bullying still remain unproven, examples of which are passive sabotage, intimidation, fraud, and unethical behaviors. Existing research has confirmed workplace bullying to be a significant trigger for deviant behavior (Sarwar et al., 2020; Zhen et al., 2021). However, as an interactive pressure from outside of the organization, it remains unclear whether customer bullying also leads to employee unethical behaviors. Further verification is needed. Moreover, it is also unclear how customer bullying affects employee unethical behaviors. This also needs further analysis.

Existing studies have mainly analyzed the relationship between customer bullying and employee behaviors from an emotional perspective (Chi et al., 2013; Walker et al., 2014), while few studies have explored the outcome effects of customer bullying from an employee cognitive perspective. The present paper predicts that frequent customer bullying causes employees to lose their sense of control and efficacy at work, resulting in job insecurity and ultimately, unethical behaviors. Based on conservation of resources theory (COR), as a negative stressor, customer bullying depletes employees’ resources, thus creating job insecurity (Almeida, 2005; Long et al., 2021). Research has also shown that job insecurity has a positive effect on unethical behaviors (Jiang et al., 2020; Sarwar et al., 2020). Therefore, job insecurity likely acts as mediator between customer bullying and unethical behaviors.

This paper posits that organizational identification is a perception of an individual’s relationship with an organization and can effectively explain individuals’ differentiated responses when faced with customer bullying (Akerlof and Kranton, 2000). According to COR, organizational identification as a psychological resource can motivate employees to work proactively and positively, thus reducing the generation of negative cognition and behaviors (Stamper and Masterson, 2002; Hu and Casey, 2021). Specifically, when individuals regard themselves as insiders in their organization, their sense of resource adequacy is potentially enhanced. To realize the value addition of resources, they are more likely to curtail the practice of unethical behaviors (Ng and Feldman, 2013). Conversely, the perception of outsider status can reduce individuals’ resource commitment to organizational or group work and even cause them to engage in unethical behaviors (Lee et al., 2015). At present, organizational identification is mainly applied to the study of organizational behaviors and to a lesser degree to the field of consumer service behaviors (Chi et al., 2013). The present paper uses organizational identification as a moderating variable to explore whether it can influence the relationship between customer bullying and unethical behaviors.

**FIGURE 1:**
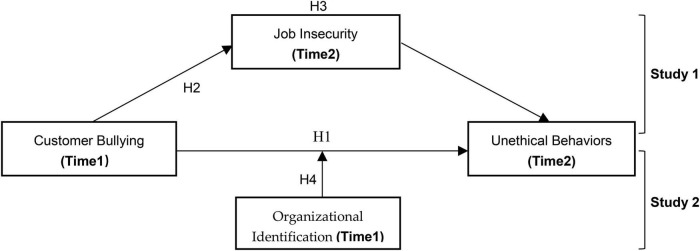
A moderated mediation model where customer bullying triggers unethical behaviors through job insecurity at low levels of organizational identification. Four hypotheses are tested using a two-wave cross-lagged survey (Time 1 and Time 2).

Based on COR, this paper explores the influence mechanism of customer bullying on employees’ unethical behavior by a dual research paradigm. Based on the above logical reasoning, this paper constructs a theoretical model as shown in Figure 1.

## Theory and Hypothesis

### Conservation of Resources Theory

Individual resources can be divided into intrinsic resources and extrinsic resources (Hobfoll, 2001). Intrinsic resources are an individual’s own internal strength, including physical, emotional, and cognitive resources. Extrinsic resources are the external strength an individual wants to obtain, e.g., organizational or social support (Jiang et al., 2020). Occupational work itself is a process of resource consumption. Individuals always face the consumption of physical and emotional resources in the process of work, but at the same time, they can obtain a supplement of resources through salary and praise (Bickerton and Miner, 2021). For employees, when the consumption and replenishment of resources are at a relatively balanced or at least tolerable level, the work pressure helps them to focus on their work and improve their work performance (Crawford et al., 2010). When work-induced resource consumption exceeds tolerable levels, the result can be physical and mental fatigue among employees, leading to reduced motivation and even aversion to work (Hobfoll, 2001; Raetze et al., 2021).

Employees often seek internal and external resources to achieve a balance of total resources, and caring behaviors such as organizational support and leadership appreciation are considered important ways to compensate for employee resources (Hobfoll et al., 2018). Rhoades and Eisenberger (2002) pointed out that organizational support has outstanding positive effects in motivating employees to work with positive emotions and increasing work engagement. However, in contrast to other industries, service employees’ direct contact with customers makes customers become the influencing factor of external resources. As the external subject with whom employees interact the most, customers provide informative feedback for employees while receiving services. If they present their feedback in a disrespectful way, this can lead to a reduction in emotional resources by causing e.g., worry and uneasiness about work. Customer behavior can cause employees to have positive or negative cognitive experiences (Raetze et al., 2021). Negative cognitive experiences not only fail to compensate for the resource consumption of the service work itself, but also lead employees to develop job insecurity (Yu et al., 2021) and can increase counterproductive behaviors (Li et al., 2012).

### Customer Bullying and Unethical Behaviors

Customer bullying is a form of workplace bullying that encompasses negative behaviors such as disrespectful, demeaning, or even verbal aggressive behaviors customers display toward employees during the consumption process (Harris and Reynolds, 2004; Skarlicki et al., 2008). Typical customer bullying is hogging a seat on a high-speed train and walking barefoot on an airplane (Wen and Liang, 2020). A survey in the hotel industry found that 86% of women experienced sexual harassment at least once at work (Zhao and Liu, 2019). Similarly, a survey in the tourism industry found that tour guides experience verbal assault by customers roughly 10 times per day over the course of their workday (Park and Kim, 2018). Customer bullying has two significant characteristics: First, bullying originates from customers, where the targets may be service employees, organizations, or other customers, and the actions include the violation of social norms, and infringement on the interests and self-esteem of others (Baranik et al., 2017; Ahmed et al., 2021). Second, regardless of whether customer bullying is intentional or unintentional, it can cause a certain amount of harm (Grandey et al., 2007). It should be noted that customer bullying is not necessarily intentional directed to harm the employee but may be caused by customer personality traits (e.g., egoism, obsessive-compulsive disorder, etc.) or poor service (Karatepe et al., 2009; Gao et al., 2019). It has been shown that customer bullying has a significant negative impact on employee emotions and performance, causing e.g., emotional exhaustion, turnover intention, and decreased job performance (Liu et al., 2012).

Unethical behaviors refer to behaviors that violate the widely accepted social norms of the group, such as aggression, deviant behaviors, negativity, intimidation, and discrimination (Kaptein, 2008; Zhang et al., 2016). Behaviors arising from the influence of extrinsic factors are also included. According to COR, individuals always try to acquire, maintain, and protect resources that are valuable to them (Hobfoll, 1989, 2001). Customer bullying can make employees feel stressed and uncomfortable, leading to resource defensive measures to address such bullying (Gao et al., 2019; Sarwar et al., 2020). It is worth noting that the protection of resources reduces the willingness of individuals to carry out positive and active behaviors, and thus increases the possibility of engaging in immoral behaviors (Sarwar et al., 2020). In the service interaction process, on the one hand, employees must spend a lot of time, emotions, labor, and other resources to meet the complex and diverse demands of customers (Brotheridge and Lee, 2002; Srivastava and Dey, 2020). On the other hand, employees gain self-esteem and a sense of efficacy from customers’ positive feedback, which is experienced as resource acquisition (Wang et al., 2011). Therefore, resource investment in services and positive feedback from customers replenish employee resources. However, negative feedback caused by customer bullying leaves employees with no resources to replenish and instead a need to devote more resources to respond to the bullying (Jiang and Lin, 2021). In this context, bullying can lead to psychological stress and negative perceptions among employees, making them more likely to engage in unethical behaviors (Muraven et al., 1998). Furthermore, continued exposure to customer bullying may trigger negative behavioral responses from employees, manifested in lowering service standards, refusal to obey organizational arrangements, and implementation of destructive behaviors (Garcia et al., 2019; Jiang and Lin, 2021). Specifically, employees who experience customer bullying often retaliate against customers by eliminating their negative emotions through disruptive service (Hoffman et al., 1995; Yue et al., 2017). According to research findings, retaliation can reduce the pain of individuals. Specifically, in the case of customer bullying, employees can experience positive emotions again by retaliating against the offender, thus reducing the negative emotions caused by the offense (Halbesleben and Bowler, 2007; Srivastava and Dey, 2020). Similarly, it has been confirmed that even though unethical behaviors have negative effects on the organization, customers, and even themselves, employees who are bullied carry out targeted retaliation against the organization or customers (Mitchell et al., 2018; Ahmed et al., 2021). Based on the above analysis, the following hypothesis is proposed.

**Hypothesis 1:** Customer bullying positively affects employees’ unethical behaviors.

### Mediating Effect of Job Insecurity

Job insecurity is treated as the perceived powerlessness to maintain desired continuity in a threatened job situation (Greenhalgh and Rosenblatt, 1984), including concerns about the threat of losing the job itself and job characteristics, such as salary, position (De Witte, 1999). Job insecurity is a subjective psychological experience of stress and has become the common psychological problems in the workplace. There are two different views on the effects of job insecurity. Some scholars view job insecurity as a challenging stressor and focus on research on positive effects, such as enhancing job performance and job initiative (Sverke et al., 2002; Hur and Perry, 2020). However, more researchers still regard job insecurity as a hindrance stressor that can negatively affect individual cognition and behavior, such as absenteeism, turnover, and counterproductive behavior (Kim and Kim, 2020; Yu et al., 2021). This study concludes that customer bullying as a workplace stressful event is likely to trigger job insecurity among service employees.

Customer bullying is an important factor triggering employees’ job insecurity. According to COR, employees experience resource depletion during interactions with bullying customers, and if resources are not compensated in a timely manner, this can impact employees’ emotions and perceptions (Sliter et al., 2012). Research findings showed that customer bullying can lead to employees’ emotional and psychological problems, such as anxiety, frustration, and fear (Harris and Reynolds, 2004). Customer bullying not only renders service personnel uncompensated for the resources consumed at work, but also further increases their job stress and causes job insecurity because of the uncontrollability of resources (Koopmann et al., 2015). Customer bullying can make employees feel helpless, thus reducing their perceived control over the work environment and tasks, thus further weakening psychological security (Ahmed et al., 2021). Furthermore, persistent customer bullying can trigger a negative perception of job value, which can lead to a loss of job confidence (Shao and Skarlicki, 2014). Therefore, chronic customer bullying can reduce employees’ sense of efficacy and meaningfulness at work and trigger job insecurity (He et al., 2019). Studies have shown that employees likely perceive customer bullying as a threat to their self-image or job performance (Weiss and Cropanzano, 1996), thus developing negative perceptions such as panic and anxiety (Jiang and Lin, 2021). Customer bullying also increases the work demands and resource requirements on frontline employees, and the resulting harsher work demands and significant resource depletion further trigger job insecurity among employees (Wang et al., 2011; Long et al., 2021). Moreover, customer bullying is generally reflected in hostile behaviors of customers toward employees’ speech and attitudes. These behaviors can lead to perceptions of job threat and trigger concerns about job stability (Yu et al., 2021). For example, research has shown that bullying behaviors such as discrimination, stigmatization, and provocation can lead to a loss of psychological resources for employees, which increases job insecurity (Knig et al., 2011). Combining the above analysis, the following hypotheses are proposed.

**Hypothesis 2:** Customer bullying positively affects employees’ job insecurity.

According to COR, when resources are depleted, threatened, or have not obtained sufficient returns, individuals will have the motivation to protect existing resources and replace lost resources (Hobfoll, 2001). The depletion of resources triggered by job insecurity prevents employees from making rational judgments and thus are more likely to engage in unethical behaviors, such as job neglect and deliberate sabotage (Mayer, 2010; Yu et al., 2021). Moreover, when employees attribute job insecurity to external factors, they need to invest self-regulatory resources to restore their mental and emotional state, thus reducing their investment of behavioral judgment resources (Mayer et al., 2012; Garcia et al., 2019). Namely, the failure of self-regulation makes employees ignore their own internal moral beliefs and may cause them to commit violations of social moral norms and engage in unethical behaviors. Studies also indicated that employees in disturbed emotional states, such as anger and anxiety, are more likely to commit aggressive behaviors (Grandey et al., 2007; Zhen et al., 2021). Similarly, employees are more likely to implement deviant behaviors to obtain psychological satisfaction under a state of job insecurity (Lee et al., 2015; Jiang et al., 2020). Based on the above analysis and combined with the derivation of Hypothesis 2, the authors suggest that job insecurity plays a mediating role between customer bullying and employee unethical behaviors. Customer bullying triggers employees’ job insecurity, leading to employees’ loss of judgment regarding ethical behaviors, thus increasing the possibility of committing unethical behaviors. Based on this, the following hypothesis is proposed.

**Hypothesis 3:** Job insecurity mediates the relationship between customer bullying and unethical behaviors.

### Moderating Effect of Organizational Identification

Customer bullying as a negative stressor can have a negative impact on employees’ perceptions and behaviors. How to reduce or eliminate the negative reactions of customer bullying on employees requires solutions from an organizational perspective. In view of this, this study introduces organizational identification as a moderating variable, and the findings can provide countermeasures for organizations to reduce the negative effects of customer bullying.

Organizational identification refers to the sense of unity employees share with their organization (Akerlof and Kranton, 2000). The higher organizational identification, the more employees will defend the organization as much as they defend themselves, and act to gain benefits for the organization (Bradler et al., 2016). Research suggested that individuals have many different identities, but individuals strive to seek and maintain positive social identities, and once established, such positive social identities create positive incentives for individual behaviors (Ng and Feldman, 2013; Hu and Casey, 2021). It has been shown that in the case of organizational identification, the outside world has a high responsibility and reputation for good deeds, and individuals will more often implement behavioral strategies that match their own identity (Rosaz et al., 2016). For example, Chazin (2014) confirmed that organizational identity will become part of an individual’s self-image, and individuals will gain self-esteem from the perception of their self-image and will strive to align their behaviors with this prototype of group identity. Ciampa et al. (2019) also showed that organizational identification triggers employees to strive toward positive social identity as well as pride of honor, which in turn makes them willing to choose positive coping strategies. For example, employees who regard themselves as insiders of an organization are more likely to maintain a positive and optimistic state, rather than choosing to commit unethical behaviors. Similarly, Bradler et al. (2016) showed that individuals who internalize organizational membership are more likely to engage in pro-organizational rather than unethical behaviors. Conversely, in the absence of organizational identification, the individual’s actual responsibility and credibility for doing good will be reduced, and the possibility of unethical behaviors will increase (Lin et al., 2015). In summary, the sense of honor and shame induced by organizational identification reduces unethical behaviors in employees. Therefore, in this study, it was hypothesized that in customer bullying dilemmas, organizational identification enhances individual psychological safety and efficacy, which reduces the engagement in unethical behaviors.

**Hypothesis 4:** Organizational identification negatively moderates the positive relationship between customer bullying and unethical behaviors.

To test the theoretical model of this study, we adopt a dual research paradigm. Study 1 adopts a questionnaire method to collect survey data at 2 time points, mainly to verify the effect of customer bullying on unethical behaviors through job insecurity. Study 2 adopted a starter experiment method to verify the moderating effect of organizational identification between customer bullying and unethical behaviors.

## Study 1: The Mediating Role of Job Insecurity Between Customer Bullying and Unethical Behaviors

### Objective

Study 1 mainly used questionnaire survey methods to test the relationship between customer bullying and unethical behaviors, and to test the mediating role of job insecurity between customer bullying and unethical behaviors.

### Sample and Sample Size Determination

Data for this study came from a panel of *N* = 260 full-time frontline employees in the Chinese hotel industry and food catering industry. All 260 employees are mainly engaged in guest room reception, restaurant reception, water bar service, dish courier, and concierge services. A total of 181 valid questionnaires were collected in this questionnaire survey (effective response rate 79.04%), including 112 male (62.21%) and 69 female (37.79%) respondents. Their average age was 25.29 years (*SD* = 6.16; range = 18–48 years). In terms of education, college education and below accounted for 64.09%, undergraduate education accounted for 26.52%, and master’s degree and above accounted for 9.39%, these sample have obtained their degrees. Specific sample demographic information is shown in Table 1.

**TABLE 1:** Sample demographics (*N* = 181).

Characteristics	Demographic	Frequency	Percent
Gender	Male	112	66.21%
	Female	69	37.79%
Age (year)	≤29	132	72.93%
	30–39	33	18.23%
	40–49	26	8.84%
	≥50	0	0%
Education	Middle school or below	12	6.63%
	High school	28	15.47%
	College	76	41.99%
	Undergraduate	48	26.52%
	Master’s degree or above	17	9.39%

### Data Collection Procedure

#### Data Collection Design

To guarantee the quality of data received, on-site collection of questionnaires was used and the human resources management department was asked to distribute the questionnaires. Questionnaires were collected through anonymous employee self-assessment and longitudinal follow-up at two time points (Time1 and Time2). All employees were numbered before the questionnaires were distributed, and then, the questionnaires were sent to respondents according to their number. In the T1 stage, the researcher distributed the first questionnaire to 260 employees. The content of the questionnaire included control variables and customer bullying, and 229 completed valid questionnaires were finally obtained (valid rate of 88.07%). After an interval of 1 month, in phase T2, based on the employee number, the researcher administered a second questionnaire to these 229 questionnaire respondents who provided valid responses in phase T1 for measuring job insecurity and unethical behaviors. After rejecting unqualified responses, 181 completed valid questionnaires were finally obtained (valid rate of 79.04%).

#### Measurement

For all measures, respondents rated the items on a 5-point scale, ranging from 1 = strongly disagree to 5 = strongly agree. The questionnaires were presented in Chinese language. Following previous guidance (Brislin, 1986), we translated English scales into Chinese, and then back-translated them, to ensure the equivalence. The scale items are shown in Table 2.

**TABLE 2:** Summary of scale items for this study.

Scale	Scale items	Literature sources
Customer bullying	Customer often say inappropriate things to me	Shao and Skarlicki, 2014
	Customer often yelled at me	
	Customer refuses to provide information (e.g., photo ID) necessary for me to do my job	
	Customer uses inappropriate gesture/body language	
	Criticized me in front of your colleagues or supervisors	
Job insecurity	I am worried about having to leave my job before I would like to	Hellgren et al., 1999
	There is a risk that I will have to leave my present job in the year to come	
	I feel uneasy about losing my job in the near future	
	My future career opportunities in (the organization) are favorable (R)	
	I feel that (the organization) can provide me with a stimulating job content in the near future (R)	
	I believe that (the organization) will need my competence also in the future (R)	
	My pay development in this organization is promising (R)	
Unethical behaviors	I will exaggerate the benefits of products or services	Mitchell et al., 2017
	I will lie to conceal my mistakes	
	To streamline the workflow, I will lower the standard of service to customers	
	I will have verbal conflicts with customers during the service	
	When not at work, I make it look like you are still at work	
	I will make up excuses for not completing my work and avoid getting into trouble	
	I’m late for work, but I won’t report it honestly	

##### Customer Bullying

Customer bullying were measured with a 5-items scales (Shao and Skarlicki, 2014). Samples include “Customer often say inappropriate things to me,” “Customer often yelled at me.” The scale’s Cronbach’s alpha was 0.866.

##### Job Insecurity

Job insecurity were measured with a 7-items scales (Hellgren et al., 1999). Samples include “I am worried about having to leave my job before I would like to” and “There is a risk that I will have to leave my present job in the year to come.” The scale’s Cronbach’s alpha was 0.914.

##### Unethical Behaviors

Unethical behaviors were measured with a 7-items scales (Mitchell et al., 2017). Samples include “I will exaggerate the benefits of products or services” and “I will lie to conceal my mistakes.” The scale’s Cronbach’s alpha was 0.916.

##### Control Variables

This paper control for several variables. Employees reported their gender (coded 1 = male; 2 = female), age (coded 1 = 29 or below; 2 = between 30 and 39; 3 = between 40 and 49; 4 = 50 and above), education experience (coded 1 = middle school or below; 2 = high school; 3 = college; 4 = undergraduate; 5 = master’s degree or above), Some scholars have pointed out that these variables can predict employee behaviors (Mitchell et al., 2018; Wen and Liang, 2020).

### Data Analysis and Results

We applied regression analysis method to test the model and casual relationships between variables. These approaches are appropriate and practical to directing and mediation effects (Clercq and Belausteguigoitia, 2016). Statistical analyses were conducted using the software SPSS 22.0 and the software AMOS. First, confirmatory factor analysis (CFA) was implemented to examine the validity and reliability of the constructs. Then descriptive statistical analysis and common method bias analysis were performed using the software SPSS 22. 0. Finally regression tests for mediating effects were performed using the program regression in the software SPSS22.0.

#### Confirmatory Factor Analysis

This study used Confirmatory factor analysis to examine the structural validity, which is designed to show how well the results obtained from the scale fit the theory assumed when designing this scale. Meanwhile, to explore whether the factor structure of the scale can be adapted to the sample data. Therefore, we used confirmatory factor analysis (*CFA*) to testify the construct validity of the major variables, namely, customer bullying, job insecurity, and unethical behaviors. The initial model’ fit index is not very well, after carrying on model modification. As shown in Table 3, the modified model yields a good fit: χ^2^/df = 2.601, RMSEA = 0.098, CFI = 0.916, TLI = 0.902, SRMR = 0.070. The factor loadings of all indicators of their constructs ranging from 0.5 to 0.91, are significant at the 0.001 level. Com-pared to the 2-factor model (χ^2^/df = 3.377, RMSEA = 0.119, CFI = 0.820, TLI = 0.799, SRMR = 0.087) and 1-factor model (χ*^2^/*df = 3.820, RMSEA = 0.130, CFI = 0.785, TLI = 0.761, SRMR = 0.101), this indicates that the discriminative validity among the three variables in this study is good, which can be used for further data analysis.

**TABLE 3:** Confirmatory factor analysis.

Model	χ^2^	*df*	χ^2^/*df*	*CFI*	*TLI*	*SRMR*	*RMSE*
Three-factor model (CB, JI, UB)	483.90	186	2.601	0.916	0.902	0.070	0.098
Two-factor model (CB + JI, UB)	634.97	188	3.377	0.820	0.799	0.087	0.119
Single factor model (CB + JI + UB)	722.28	189	3.820	0.785	0.761	0.101	0.130

*CB, Customer bullying; JI, Job insecurity; UB, Unethical behaviors.*

#### Single Factor Analysis

The variable data in this study are all self-rated by employees, which may lead to common method bias and false correlation between constructs, and thus affecting the accuracy and validity of data analysis. Therefore, Harman single-factor test in this study was adopted to test the common method bias of data. Specifically, exploratory factor analysis without rotation was conducted, and the results showed that there were 3 common factors with eigenvalues greater than 1, and the first common factor only explained 21.62% of the overall variation, which was lower than the 40% judgment standard (Podsakoff et al., 2003). This indicates that there is no serious common method bias in the data of this study.

#### Descriptive Statistics and Correlations

Table 4 displays means, standard deviations and correlations of the major variables and control variables. As expected, customer bullying was significantly positively correlated with employees’ unethical behaviors (*r* = 0.579, *p* < 0.01), and positively correlated with job insecurity (*r* = 0.648, *p* < 0.01). Job insecurity and unethical behaviors are significantly positively correlated (*r* = 0.705, *p* < 0.01). The above results provide preliminary support for the hypothesis of this study.

**TABLE 4:** Means, standard deviations, correlations among the study variables.

Variable	Mean	SD	CR	AVE	MSV	1	2	3
1. Customer bullying	3.57	0.64	0.906	0.661	0.342	**1**		
2. Job insecurity	3.79	0.64	0.932	0.663	0.318	0.648**	**1**	
3. Unethical behaviors	3.44	0.72	0.931	0.605	0.406	0.579**	0.705**	1

SD, standard deviation; CR, combined reliability; AVE, average variance extracted; MSV, Maximum shared variance. Reliabilities are listed in parentheses. **Represent p < 0.01.

*The meaning of the bold values: Mean = average value; SD = standard deviation; CR = combined reliability; AVE = average variance extracted; MSV = Maximum shared variance.*

Moreover, Table 2 analyzes the reliability and validity of the variables. The results show that the composite reliability (CR) is acceptable (>0.7), the convergent validity and discriminant validity are also acceptable (AVE > 0.5 > MSW). This indicates that all three variables have good reliability and validity.

#### Hypothesis Testing

First, the direct effects of customer bullying on unethical behaviors and the indirect effects through job insecurity were tested. Hypothesis 1 predicts that customer bullying is related to unethical behaviors. As Table 5 shows, the correlation between customer bullying and unethical behaviors is significant (beta = 0.651, *p* < 0.001), implying that the association between customer bullying and unethical behaviors is significant. This supports Hypothesis 1.

**TABLE 5:** Regression results for direct and mediating effects.

Variable	Job insecurity				Unethical behaviors			
	Model 1		Model 2		Model 3		Model 4	
	Beta	SE	Beta	SE	Beta	SE	Beta	SE
Sex	0.002	0.081	0.014	0.097	0.012	0.072	0.013*	0.071
Age	0.092*	0.046	0.096*	0.056	0.016	0.041	0.020	0.041
Education	–0.078	0.072	–0.082	0.087	–0.003	0.063	−0.017*	0.064
Customer bullying	0.648***	0.302	0.651***	0.071			0.116***	0.069
Job insecurity					0.600***	0.053	0.524***	0.069
*F*	32.176		22.281		75.273		61.471	
△*R*^2^	0.441***		0.353***		0.649***		0.655***	

*Represent p < 0.05, **represent p < 0.01, ***represent p < 0.001.

Hypothesis 2 predicts that customer bullying is related to job insecurity, as Table 5 shows, we found that the correlation between customer bullying and job insecurity is significant (beta = 0.648, *p* < 0.001). This implies that the association between customer bullying and job insecurity is significant, which supports Hypothesis 2.

Hypothesis 3 predicts that job insecurity mediates the relationship between customer bullying and unethical behaviors. The result shows that customer bullying was positively related with both job insecurity (beta = 0.648, *p* < 0.001) and unethical behaviors (beta = 0.651, *p* < 0.001). Job insecurity was also positively related with unethical behaviors (beta = 0.600, *p* < 0.001). When considering these variables together, the coefficient between customer bullying and unethical behaviors decreased (beta = 0.116, *p* < 0.001), and job insecurity was also positively related with unethical behaviors (beta = 0.524, *p* < 0.001). This indicates that job insecurity plays a partial mediating role in the relationship between bullying on unethical behaviors, which supports Hypothesis 3.

The empirical results of this study verified Hypotheses 1, 2, and 3, indicating that the positive relationship between customer bullying and unethical behaviors was verified, and that job insecurity partially mediates the relationship between customer bullying and unethical behaviors. Through Study 1, a causal relationship between customer bullying and unethical behaviors could be established. It is worth noting that Study 1 also has certain limitations, i.e., it has not explored the boundary conditions in the influence mechanism of customer bullying. To further enhance the external validity of the theoretical model, this paper uses experimental methods to confirm the conclusion of Study 1 in Study 2 and based on the results, the moderating effect is tested.

## Study 2: The Moderating Effect of Organization Identification Between Customer Bullying and Unethical Behaviors

### Objective

This experiment had two objectives. First, to provide further support for the conclusion of Hypothesis 1 through experimental analysis. Second, in this experiment, the organizational identification measurement items proposed by Cohn et al. (2017) are used. These are defined as “When I think of myself, I often think of myself as a member of the organization,” and draws on Cohn and Marechal’s induction method to study the impact of customer bullying and identification interactions on unethical behaviors.

### Sample and Sample Size Determination

The experimental subjects in this study were sourced from six financial institutions (i.e., banks and securities companies) in China. A field experiment was conducted, before the experiment was formally started. The purpose and overview of this experiment were first introduced with the human resource manager of each company, and 232 full-time frontline employees were recruited as subjects on a voluntary participation basis. These employees are mainly engaged in cash business, treasury business, and corporate business. During the experiment, 20 subjects canceled the experimental agreement for unknown reasons, while a total of 212 subjects completed the experiment. Each participant received a nice souvenir after the experiment. Participants included 98 male (46.23%) and 114 female (53.77%). Their average age was 32.62 years (SD = 7.81; range = 21–56 years). In terms of education, college education and below accounted for 2.36%, undergraduate education accounted for 84.91%, master’s degree and above accounted for 12.73%. These sample have obtained their degrees.

### Experimental Design and Procedure

#### Experimental Design

A total of 212 financial practitioners were invited to participate in this study (46.23% male, mean age = 32.62 years), and all participants were informed that they were participating in a study on customer bullying behaviors. Participants were randomly assigned to a single-factor intergroup design (identification group vs. control group), namely, the identification group with high organizational identity and the control group with low organizational identity, both with 106 participants. Specifically, the investigators numbered all participants based on the order of arrival (numbers 1–212). Participants with odd numbers were categorized as the identification group and those with even numbers were categorized as the control group. After entering the laboratory, participants first listened to the investigator who introduced the experimental process and requirements. Then, the experiment carried out, and the test scale and personal statistical information were filled in. Finally, the participants were asked about the purpose of the experiment, were thanked, and received souvenirs.

#### Experimental Procedure

The experimental manipulation used organizational identification by induction, which is widely used for experimental manipulation in social psychology and organizational behaviors. Specifically, in the manipulation group (i.e., the identification group), employees in the manipulation group were asked to answer six questions related to their job, such as “the name of your organization” and “your best friend at your organization.” Employees in the control group were asked to answer six questions not related to their job, such as “your favorite food” and “your favorite exercise.”

After the six questions were completed by the control group and the identification group, participants were asked to confirm the questions he/she had answered. After the manipulation, participants were asked to continue to complete an unrelated descriptive task (i.e., inviting subjects to describe what they usually do on weekends) as a filler task (Berger et al., 2008). Subsequently, participants were asked to fill in the organizational identification measure, i.e., “When I think of myself, I often think of myself as a member of the organization,” where 1 = “not at all” and 5 = “completely.”

When the organizational identification measures were completed, all participants were asked to fill out the customer bullying and unethical behaviors scales as well as provide demographic information. The study instruments were consistent with Study 1, the customer bullying Cronbach’s alpha was 0.926, and the unethical behaviors Cronbach’s alpha was 0.718.

### Data Analysis and Results

The independent sample *T*-test results showed that the identification level (*n* = 106, *M* = 3.525, *SD* = 0.589) reported by the manipulation group (identification group) was significantly higher than the identification level reported by the control group (*n* = 106, *M* = 2.81, *SD* = 0.266). There is a significant difference between the two groups (*t* = 25.314, *p* < 0.001). In conclusion, this study was effective in manipulating identification.

#### Hypothesis Testing

The regression method was used to test for direct and moderating effects, and the results are shown in Table 6. Model 1, customer bullying has a significant positive effect on unethical behaviors (Beta = 0.555, SE = 0.042, *t* = 13.303, *p* < 0.001), and Hypothesis 1 was further tested.

**TABLE 6:** Regression results for direct and moderating effects.

Variable	Unethical behaviors					
	Model 1			Model 2		
	Beta	SE	*t*	Beta	SE	*t*
Sex	–0.04	0.075	–0.538	–0.045	0.074	–0.606
Age	–0.061	0.045	–1.354	–0.058	0.045	–1.294
Education	0.066**	0.04	1.673	0.062*	0.039	1.579
Customer bullying	0.555***	0.042	13.303	0.556***	0.129	13.42
Organizational identification	−0.076*	0.041	–1.862	−0.081*	0.118	–2.001
Customer bullying × identification				−0.078**	0.044	–2.136
*F*	48.092			66.033		
△R2	0.472***			0.488***		

*Represent p < 0.05, **represent p < 0.01, ***represent p < 0.001.

**FIGURE 2:**
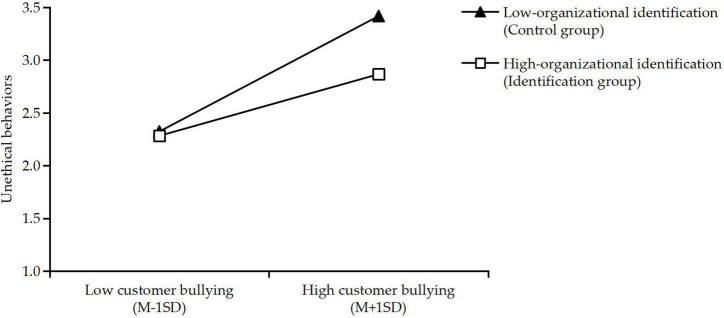
Showing the interactive effects of customer bullying and organizational identification on unethical behaviors. Slope for low organizational identification (β = 0.81, *p* < 0.001); slope for high organizational identification (β = 0.66, *p* < 0.01). Low-organizational identification, control group; high-organizational identification, identification group. Low customer bullying, *M* - 1*SD* (one standard deviation below the mean). High customer bullying, *M* + 1*SD* (one standard deviation above the mean).

Model 2 shows that unethical behaviors were used as dependent variable, organizational identification (0–1 variable, 0 represents no organizational identification and 1 represents organizational identification) and the interaction term customer bullying × organizational identification were used as independent variables, and gender, age, and education were used as control variables in a regression analysis using SPSS 22.0. The results show that the analysis model is significant (*F* = 66.033, *p* < 0.001), and there is no multicollinearity problem (*VIFs* < 2). The regression coefficient of customer bullying on unethical behaviors is significant (Beta = 0.555, SE = 0.042, *t* = 13.303, *p* < 0.001), the influence of organizational identification on unethical behaviors is significant (Beta = −0.081, SE = 0.118, *t* = −2.001, *p* < 0.05), and the regression coefficient of customer bullying × organizational identification is significant (Beta = −0.078, *SE* = 0.044, *t* = −2.136, *p* < 0.01). Hypothesis 4 is supported. To better demonstrate the moderating effect of organizational identification, a simple slope analysis was carried out, demarcating between high (*M* + *SD*) and low (*M* - 1*SD*) levels of customer bullying. As shown in Figure 2, when organizational identification is high (identification group), the positive influence of customer bullying on unethical behaviors is weakened (simple slope = 0.66, *p* < 0.01); in contrast, when organizational identification is low (control group), the positive influence of customer bullying on unethical behaviors is strengthened (simple slope = 0.81, *p* < 0.001). This further supports the conclusion of Hypothesis 4. Further, gender and age were not significantly different between Model 1 and Model 2, while educational background was significantly different between Model 1 and Model 2. Hence, Hypothesis 4 was supported.

The results of this study suggest that employee organizational identification moderates the relationship between customer bullying and unethical behaviors. In the context of organizational identification, even if the level of customer bullying is high, the unethical behaviors of employees will not be very high. But in the context of no organizational identification, customer bullying can more easily lead to unethical behaviors by employees. The research conclusion is consistent with the hypothesis.

## Discussion

Based on this analysis, the positive effect of customer bullying on unethical behaviors is confirmed. Specifically, customer bullying depletes employees’ emotional and psychological resources, and thus leads to employees’ retaliation in the form of committing unethical behaviors. This study confirms that customer bullying can lead to unethical behaviors. Moreover, customer bullying can cause employees to worry and feel uneasy about the continuity of their current jobs, and this negative perception often leads to job insecurity. Under the influence of job insecurity, employees are more inclined to carry out unethical behaviors in retaliation against organizations. Therefore, this study confirms that job security mediates the relationship between customer bullying and unethical behavior. Finally, employees’ identification with the organization as an intrinsic resource can compensate for the depletion of employees’ resources in dealing with customer bullying, thus reducing their negative perceptions and unethical behavior.

### Theoretical Contributions

This study confirms that there is a positive relationship between customer bullying and unethical behaviors, meaning that when employees encounter customer bullying, they tend to exhibit unethical behaviors. Customer bullying is a kind of interactive stress, and its impact on unethical behaviors is consistent with the conclusions of previous work stress research (Ahmed et al., 2021). Based on COR, this study found that customer bullying consumes a large amount of employees’ psychological resources, which will lead employees to retaliate through unethical behaviors to achieve a psychological balance. Moreover, previous research on unethical behavior mainly focused on the field of organizational behavior (Koopmann et al., 2015; Yue et al., 2017), but it was rarely applied to the field of consumer industry. Therefore, this study explores the relationship between customer bullying and unethical behaviors and expands the perspective of unethical behavior research.

This study confirms that customer bullying can trigger feelings of job insecurity in employees. Customer bullying creates negative work experience and resource depletion, which will cause employees to worry about job stability and control. This finding is consistent with previous research on the effects of customer bullying on employees’ emotions (Koopmann et al., 2015), which also concluded that customer bullying leads to job insecurity. Based on COR, responding to customer bullying requires more resources, which leads to a reduced sense of control over employees’ resources, and thus triggers concern about their jobs. This paper explores the mechanism of customer bullying from a cognitive perspective. On the one hand, it expands the theoretical perspective of customer bullying, while on the other hand, it also confirms that negative work stress can trigger negative perceptions.

This study constructs and tests the mechanism of how customer bullying influences employee unethical behaviors through job insecurity. At present, there are only few relevant studies on customer bullying, which mainly focused on the impact of customer bullying on work attitudes and behaviors (Fullerton and Punj, 1993; Walker et al., 2014). The present study used job insecurity as mediating variable, which is in line with the uncertain employment environment background induced by the flexible economy and COVID-19. The research content meets the requirements of contemporary theoretical studies. Previous studies have mainly explored customer bullying mechanisms from emotional and resource perspectives (Walker et al., 2014; Baranik et al., 2017), while few studies have explored the influence mechanisms between customer bullying and employee behavior from a cognitive perspective. Moreover, research that analyzes the relationship between customer bullying, job insecurity, and unethical behaviors is practically missing. Therefore, this study opens the “black box” of the influence mechanism of customer bullying from a cognitive perspective, which compensates for the shortcomings of previous research perspectives.

This research also constructs a moderation model to test the moderating effect of organizational identification between customer bullying and unethical behaviors. Previously, organizational identification was mainly employed in the field of organizational behaviors (Wang et al., 2011; Sliter et al., 2012), but not in the field of consumer services. In this study, organizational identification as a moderating variable has two meanings. First, it helps to clarify the boundary conditions of customer bullying affecting unethical behaviors. This study confirms that individual organizational identification can mitigate the negative effects of customer bullying on employees, a finding that enriches the knowledge on factors influencing customer bullying. Second, the findings of this study not only clarify that organizational identification plays an important role in reducing employees’ unethical behavior, but also highlight the positive impact of organizational identification in improving employees’ sense of security.

Furthermore, the theoretical model of this study involves variables of multiple disciplines. This study constructs a multidisciplinary integrated theoretical model of consumer behavior, organizational behavior, and psychology, which provides new ideas for subsequent customer bullying research. Moreover, this study expands the methodological perspective of customer bullying research. Previous research on the outcome effects of customer bullying has been dominated by questionnaire analysis (Halbesleben and Bowler, 2007), with little implementation of experimental methods for analysis and validation. Study 2 used an experimentally initiated approach to manipulate the identification of participating employees with the goal to verify that identification negatively moderates the effect of customer bullying on unethical behaviors. Finally, this study extended COR theory (Hobfoll, 2001, 2002) by purporting organizational identification as an individual resource which helps to buffer the relationship between customer bullying and unethical behaviors. In doing so, this paper responds to recent calls to include other resources which can diminish the harmful consequences of workplace bullying in the health care industry of China (Cheng et al., 2019; Wen and Liang, 2020).

### Managerial Implications

The findings of this study have the following important implications. First, the power asymmetry between customers and employees is a major factor in the recent growth of customer bullying. From the customer’s point of view, organizations need to develop ethics in their interactions with service personnel while pursuing customer satisfaction to reduce instances of customer bullying of service providers. From an organizational point of view, managers should place employees and customers at an equal level. While respecting the needs of customers, they must also protect the rights and interests of employees, thus creating a positive interactive service situation. From the perspective of employees, the organization should arrange interpersonal skills training and stress training for front-line employees, thus helping them to deal with the harm caused by customer bullying.

Second, job insecurity plays a mediation role between customer bullying and unethical behaviors. Therefore, when employees suffer from customer bullying, organizations should provide timely cognitive and emotional counseling to reduce the negative effects of job insecurity. Moreover, organizations should make their jobs more secure by providing stable employment opportunities for service workers.

Finally, organizational identification negatively moderates the relationship between customer bullying and unethical behaviors. Managers should guide employees to perform identity recognition and improve the level of their internal perception of identity, thereby enhancing their own perception of responsibilities and obligations. Moreover, through training, welfare policies, and value implantation, organizations can increase employees’ perception of insider status and increase their emotional attachment. These measures can reduce the level of unethical behaviors employees commit.

### Limitations and Future Research

This study has several limitations. First, the sample comes from a single region and industry, which may lead to a lack of universality in the analysis results. Future research can expand the scope of research in regions and industries, and obtain more universal conclusions based on the empirical analysis of a large sample. Second, this study only considers the mediating role of job insecurity and the moderating role of organizational identification, without considering other mediating and moderating variables, which limits the explanatory power and completeness of the theoretical model to a certain extent. During the service interaction, employees’ unethical behavior may arise in relation to other factors, such as the supervisor-subordinate guanxi, work intensity and work complexity. Further research is needed to further refine the theoretical model and support the application of the theory in practice. Finally, we examined the effect of customer bullying on employees’ negative behavioral responses. However, it is worth noting that as a stressful event, customer bullying is likely to have positive effects, such as stimulating work motivation, autonomy, and service performance. Hence, future research can explore whether customer bullying has a positive impact, so as to improve the theoretical research on the impact effect of customer bullying.

## Conclusion

Based on the COR, This study proposes a theoretical model of the influence mechanism of customer bullying on employee unethical behaviors, and discusses the mediating effect of job insecurity and the moderating effect of identification. Using front-line employees in the service industry as data samples, the analysis results found that customer bullying has a positive impact on job insecurity and unethical behaviors. Job insecurity partially mediating the relationship between customer bullying and unethical behaviors. In the process of interactive service, customers’ bullying behavior toward employees will make employees experience job insecurity and then lead to the implementation of unethical behavior. Furthermore, the organizational identification weakens the positive relationship between customer bullying and unethical behaviors. In other words, organizational identification can reduce the negative impact of customer bullying on employees. Hence, managers and human resources professionals should improve employee job security and organizational identification that enhance their psychological tolerance to reduce the occurrence of unethical behaviors.

## Data Availability Statement

The raw data supporting the conclusions of this article will be made available by the authors, without undue reservation.

## Ethics Statement

The studies involving human participants were reviewed and approved by the Ethics Committee of South China University of Technology. The patients/participants provided their written informed consent to participate in this study. Written informed consent was obtained from the individual(s) for the publication of any potentially identifiable images or data included in this article.

## Author Contributions

All authors made a significant contribution to the work reported, whether that is in the conception, study design, execution, acquisition of data, analysis, and interpretation, or in all these areas, took part in drafting, revising or critically reviewing the article, gave final approval of the version to be published, have agreed on the journal to which the article has been submitted, and agreed to be accountable for all aspects of the work.

## Conflict of Interest

The authors declare that the research was conducted in the absence of any commercial or financial relationships that could be construed as a potential conflict of interest.

## Publisher’s Note

All claims expressed in this article are solely those of the authors and do not necessarily represent those of their affiliated organizations, or those of the publisher, the editors and the reviewers. Any product that may be evaluated in this article, or claim that may be made by its manufacturer, is not guaranteed or endorsed by the publisher.
